# Leveraging artificial intelligence for collaborative care planning: Innovations and impacts in shared decision-making – A systematic review

**DOI:** 10.1515/med-2025-1232

**Published:** 2025-08-29

**Authors:** Gianmarco Di Palma, Roberto Scendoni, Anna De Benedictis, Vittoradolfo Tambone, Francesco De Micco

**Affiliations:** Research Unit of Bioethics and Humanities, Department of Medicine and Surgery, Università Campus Bio-Medico di Roma, Roma, 00128, Italy; Department of Clinical Affair, Fondazione Policlinico Universitario Campus Bio-Medico, Roma, 00128, Italy; Department of Public Health, Experimental and Forensic Medicine, University of Pavia, Pavia, Italy; Department of Law, University of Macerata, Macerata, 62100, Italy; Research Unit of Nursing Science, Department of Medicine and Surgery, Università Campus Bio-Medico di Roma, Rome, Italy

**Keywords:** advanced care planning, shared decision making, artificial intelligence

## Abstract

**Introduction:**

Advance care planning is a critical process that brings patients, their families, and healthcare providers together to set goals and outline preferences for future medical treatments, especially when chronic or terminal illnesses are involved. Recently, artificial intelligence has begun playing a key role in shared decision making, offering personalized recommendations based on detailed data analysis to help refine treatment decisions.

**Objective:**

This review explores Artificial Intelligence’s role in shared decision making, noting its potential to enhance treatment precision, reduce the workload for healthcare providers, and empower patients to engage more actively in their cares.

**Methods:**

The systematic review was conducted using the The Preferred Reporting Items for a Systematic Review and Meta-Analysis Statement 2020 guidelines to ensure a comprehensive and transparent approach. We utilized the online tool Rayyan for screening and selection of relevant studies.

**Results:**

The review highlights the importance of transparency and clinician involvement to ensure that artificial intelligence remains a supportive, rather than dominant, element in patient care. Emphasizing the human aspect of decision-making is essential, as is fostering a collaborative approach between artificial intelligence and healthcare professionals.

**Conclusion:**

Artificial intelligence holds promise in transforming shared decision making, ongoing research must address these implementation challenges to secure its ethical and patient-centered use in healthcare.

## 1 Introduction

Advance care planning (ACP) is a process of communication between the patient, family members, and healthcare professionals, which supports patients in considering their preferences for future care in the event of illness progression or a significant health change that may affect decision-making ability. A primary objective of ACP is to honor patient autonomy by allowing them to express their wishes in advance, ensuring these decisions are followed even if the patient loses capacity [1]. Engagement fosters communication among patients, families, and the healthcare team, aligning therapeutic decisions with personal care goals [2]. This can also prevent potential family-doctor conflicts regarding end-of-life decisions by providing clear guidance on patient preferences [3]. A central component of ACP is the advanced directive discussion, based on repeated conversations as preferences evolve and require regular updates [2]. Autonomy is prioritized, allowing patients to formalize their choices in written documents like advance directives or living wills [4]. ACP also includes a multidisciplinary component – nurses, doctors, psychologists, and social workers provide comprehensive decision-making support [3].

The doctor-patient relationship is founded on four core elements: mutual recognition, trust, loyalty, and respect. During the SARS-CoV-2 pandemic, the imposed lockdown catalyzed technologies that bridge interpersonal distances, also influencing healthcare through the digitization and virtualization of health and social services. Researchers characterize the post-pandemic era as one marked by digitalization and artificial intelligence (AI), with healthcare systems increasingly using digital AI platforms in service delivery [5,6].

An emerging area of research focuses on integrating AI in shared decision making (SDM) to enhance medical decision-making. By processing large amounts of clinical data, AI can provide personalized recommendations based on accurate predictions for treatment outcomes, aiding both informed and targeted decision-making. This method serves as clinical decision support, with AI algorithms guiding doctors in identifying tailored therapeutic interventions based on historical data and predictive outcomes [7]. As a result, managing complex information becomes simpler, allowing doctors to focus on compassionate conversations with patients [8]. Additionally, AI tools can integrate clinical, biological, and genetic data to infer the likelihood of success for different treatments, helping patients identify the best options [9]. This systematic review set out to explore how AI is currently applied to support SDM in clinical settings. The aim was to identify and analyze studies that examine AI’s role in enhancing the quality of decision-making processes, increasing patient satisfaction, promoting transparency, enabling personalized care, and assisting healthcare professionals in clinical decision-making.

## 2 Materials and methods

**Figure 1:**
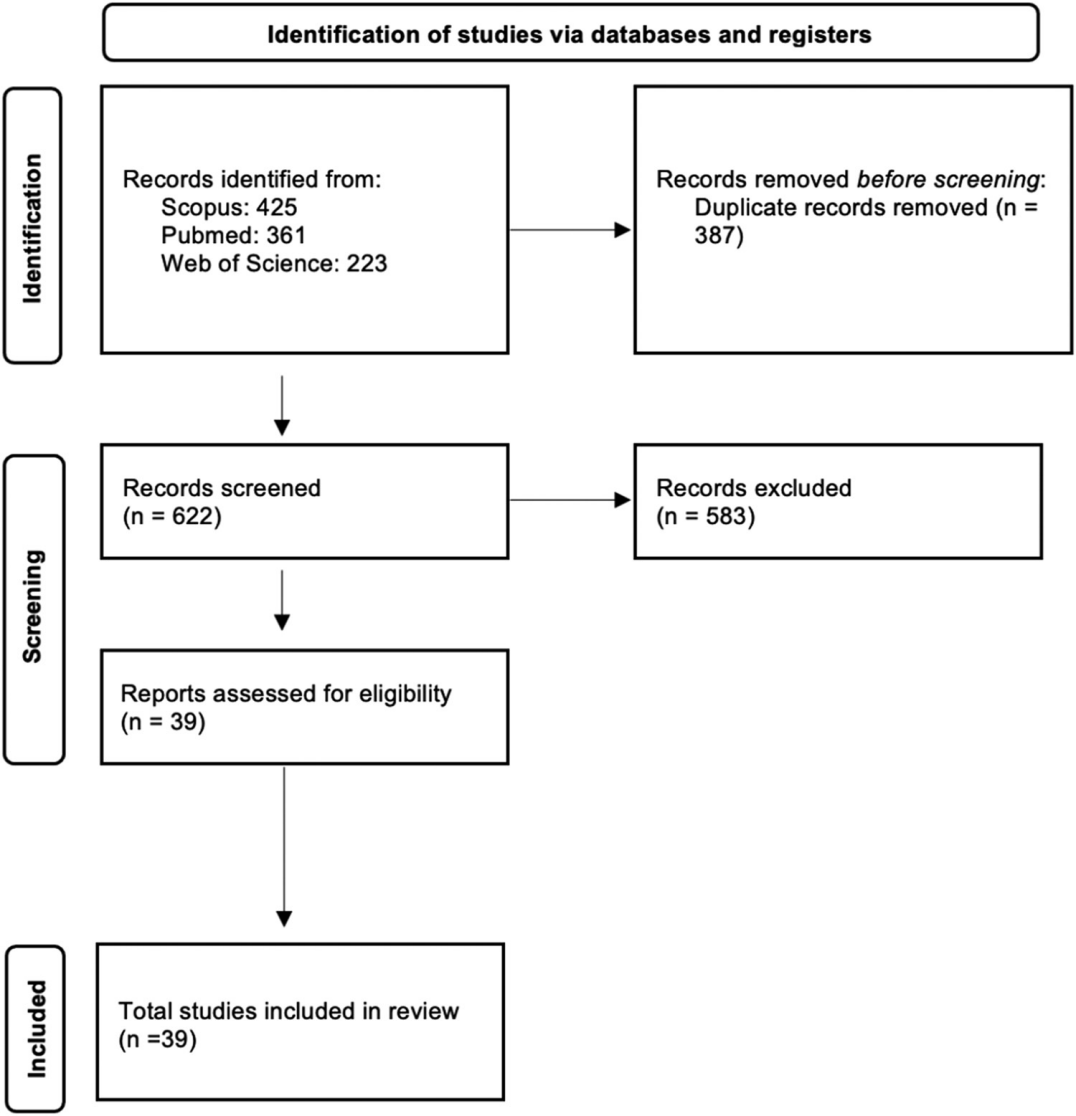
Representation of the selection and review of papers.

The methodology of this review was based on guidelines from The Preferred Reporting Items for a Systematic Review and Meta-Analysis of Diagnostic Test Accuracy Studies (PRISMA-DTA) [10]. A systematic literature review on the use of AI in the routine aspect of ACP was undertaken. Electronic databases PubMed, Scopus, and Web of Science were searched using the following terms: “((shared decision) OR (shared decision making)) AND ((artificial intelligence) OR (AI) OR (machine learning) OR (deep learning) OR (artificial neural network)).” Studies were included if they contained original research or protocols concerning the use of AI in the context of SDM. Eligible studies had to address outcomes such as improvement in decision quality, patient satisfaction, transparency, personalization of care, or support for the clinical decision-making process. Articles were excluded if they addressed AI in healthcare without reference to SDM, if they described generic digital tools within the scope of ACP without AI involvement, or if they were published in languages other than English. The search took place on March 17, 2024, yielding a total of 1,009 articles. Duplicate entries were removed using the Rayyan tool, leaving 622 articles. After an initial review, 583 were excluded for not meeting the inclusion criteria (Figure 1).

## 3 Results

This systematic review highlights that while AI systems in SDM are still in their early developmental stages, their potential for significant impact is already evident as also inferable from the main findings represented in Table 1. For example, Afsar [11] developed a versatile AI health bot to assist doctors and patients in treatment selection, communication, and workload management, offering patients more autonomy over their health. The system provides personalized recommendations based on data and established guidelines. In a related study, Begley *et al.* [12] examined “inherited defeaters” (biases that influence decision-making in obstetrics), proposing strategies to mitigate these biases and improve AI reliability in such contexts. Other research demonstrates AI’s promise across various medical fields: Brown *et al.* [13] used machine learning models to prevent cardiovascular toxicity in cancer patients, applying patient similarity-based methods within SDM. Chen *et al.* [14] designed a protocol for a randomized trial evaluating a web-based tool to assist women in deciding between vaginal birth and cesarean section, focusing on satisfaction, anxiety, and delivery outcomes. Crook *et al.* [15] reviewed AI systems providing information on hand surgery, noting the accessibility benefits but also the potential risks of misinformation, underscoring the need for professional oversight. Dantas and Nogaroli [16] analyzed the ethical and legal implications of AI and robotics in informed consent, stressing the importance of updating liability frameworks. De Vries *et al.* [17] introduced the Boosting framework to enhance SDM in health informatics, promoting transparency and improving the patient-clinician relationship. Swamy and Grotegut [18] explored digital tools designed to support women considering labor after cesarean, presenting a balanced risk-benefit analysis. Gould *et al.* [19] examined patient perspectives on AI’s role in predicting knee surgery risks, finding mixed opinions on AI’s reliability compared to traditional consultations. Greco and Picozzi [20] discussed AI’s impact on the doctor-patient relationship, highlighting not only diagnostic improvements but also potential drawbacks to patient autonomy. Hassan *et al.* [21] developed a guide for creating AI prediction models, while an associated study [22] explored clinicians’ and patients’ views, revealing both trust in AI and concerns about reduced personal interaction. Further studies show AI’s expanding role in SDM beyond diagnostics: Holm [23] advocated for SDM frameworks that go beyond diagnostic accuracy, while Höppchen *et al.* [24] examined AI’s role in cardiac rehabilitation. Jang *et al.* [25] proposed an interpretable model for decision-making in hip arthroplasty. Jayakumar *et al.* [26] showed that AI improved patient satisfaction in knee osteoarthritis decision-making compared to traditional methods. Jin *et al.* [27] introduced a visualization tool to support responsible antibiotic prescribing. Jayakumar *et al.* [28] explored how generative AI and large language models could act as catalysts for value-based healthcare, emphasizing their potential to enhance decision-making processes and improve patient outcomes. Additional studies include Kruser *et al.* [29], focusing on improving documentation in SDM within intensive care, Karaa’s [30] insights into AI in dermatology, and Kather’s [31] cautious approach to AI in oncology. Khosravi *et al.* [32] reviewed ethical considerations in healthcare AI, while Kokkinakis *et al.* [33] explored AI’s potential in surgical risk prediction. Davidson and Boland [34] focused on AI’s role in pregnancy-related drug decision-making, and Li *et al.* [35] examined AI’s impact on pediatric SDM, identifying challenges with integration. Other contributions emphasize maintaining the doctor-patient relationship [36], integrating values-based AI [37], and ensuring transparency in AI-assisted decisions [38]. Nair *et al.* [39] advocated for the development of accessible SDM tools, while Pedersen *et al.* [40] presented the “PROPOSE” model to enhance SDM for spinal stenosis care. Pinton [41] discussed both the benefits and challenges of AI in managing inflammatory bowel disease. Rahimi *et al.* [42] explored AI’s potential to improve patient engagement and Ran *et al.* [43] focused on transparency in assistive AI development. The 4D PICTURE project by Rietjens *et al.* [44] implemented data-driven pathways to optimize SDM in cancer care. Sauerbrei *et al.* [45] recommended balancing AI’s utility with a patient-centered approach, while Topaz *et al.* [46] highlighted voice recognition’s potential to enhance SDM in primary care settings. Finally, Tretter *et al.* [47] advocated balancing AI’s benefits with ethical standards, Xu *et al.* [48] examined machine learning applications in breast reconstruction surgery, and Yung *et al.* [49] affirmed AI’s supportive role in oncology SDM, though gaps in training remain.

**Table 1:** Overview of reviewed studies

Title	Topic	Type of study	Main conclusion
Intelligent Multi-Purpose Healthcare Bot Facilitating Shared Decision Making [11]	Multipurpose chatbot to support SDM	Technical development studio	AI bot improves doctor-patient communication and supports shared decision making
Shared Decision-Making and Maternity Care in the Deep Learning Age: Acknowledging and Overcoming Inherited Defeaters [12]	SDM and motherhood in the era of deep learning	Theoretical/critical article	Highlights inherited obstacles to SDM with AI; proposes strategies to overcome them
Patient Similarity and Other Artificial Intelligence Machine Learning Algorithms in Clinical Decision Aid for Shared Decision-Making in the Prevention of Cardiovascular Toxicity (PACT): A Feasibility Trial Design [13]	AI for cardiovascular toxicity prevention	Feasibility study	The use of AI (patient similarity, machine learning) can support clinical SDM in trials
An Innovative Web-Based Decision-Aid about Birth after Cesarean for Shared Decision Making in Taiwan: Study Protocol for a Randomized Control Trial [14]	AI-based decision aid for delivery after cesarean section	RCT protocol	Evaluates impact of a web-based tool on SDM in pregnancy
Evaluation of Online Artificial Intelligence-Generated Information on Common Hand Procedures [15]	Information quality AI online about orthopedic procedures	Evaluative study	AI provides useful, but not always accurate SDM info
The Rise of Robotics and Artificial Intelligence in Healthcare: New Challenges for the Doctrine of Informed Consent [16]	AI and informed consent	Doctrinal discussion	AI challenges traditional concept of consent; new legal paradigms needed
Fostering Shared Decision Making with Health Informatics Interventions Based on the Boosting Framework [17]	IT framework to promote SDM	Methodological study	Use of the Boosting Framework can enhance physician-patient interaction
Can a Structured, Electronic Approach to Shared Decision-Making Increase Attempted Trial of Labor? [18]	SDM structured electronic vaginal delivery system	Letter/short study	Structured electronic approach increases vaginal delivery attempts after cesarean
Patients’ Views on AI for Risk Prediction in Shared Decision-Making for Knee Replacement Surgery: Qualitative Interview Study [19]	AI for risk prediction in knee replacement	Qualitative study	Patients value AI in SDM but demand transparency
Understanding the Impact of Artificial Intelligence on Physician-Patient Relationship: A Revisitation of Conventional Relationship Models in the Light of New Technological Frontiers [20]	AI and the doctor-patient relationship	Theoretical article	AI forces a rethinking of traditional relational models
Road Map for Clinicians to Develop and Evaluate AI Predictive Models to Inform Clinical Decision-Making [21]	Guidelines for developing AI-based prediction models	Methodological paper/Framework development	The development and evaluation of AI predictive models in clinical decision-making should follow a structured road map, emphasizing transparency, validation, and integration into clinical workflows for improved patient outcomes
Clinicians’ and patients’ perceptions of the use of AI decision aids to inform shared decision making: a systematic review [22]	Clinicians’ and patients’ views on AI: Trust and reduced personal interaction	Qualitative study (opinions/perceptions)	Both clinicians and patients recognize the potential of AI decision aids to enhance shared decision-making, but concerns regarding trust, understanding, and integration into clinical practice must be addressed for effective use
Handle with Care: Assessing Performance Measures of Medical AI for Shared Clinical Decision-Making [23]	Need for SDM frameworks beyond diagnostic accuracy	Conceptual/theoretical study	Medical AI for shared clinical decision-making requires careful evaluation of its performance measures to ensure it aligns with ethical standards and enhances patient outcomes
Targeting Behavioral Factors with Digital Health and Shared Decision-Making to Promote Cardiac Rehabilitation-a Narrative Review [24]	AI’s role in cardiac rehabilitation	Applied/empirical study	Digital health tools, combined with shared decision-making, can effectively target behavioral factors to enhance cardiac rehabilitation, though their success depends on patient engagement and personalization
An Interpretable Machine Learning Model for Predicting 10-Year Total Hip Arthroplasty Risk [25]	Interpretable AI model for decision-making in hip arthroplasty	Model development and validation	An interpretable machine learning model effectively predicts the 10-year risk of total hip arthroplasty, aiding clinicians in making informed decisions for long-term patient care
Comparison of an Artificial Intelligence-Enabled Patient Decision Aid vs Educational Material on Decision Quality, Shared Decision-Making, Patient Experience, and Functional Outcomes in Adults with Knee Osteoarthritis: A Randomized Clinical Trial [26]	Improved patient satisfaction in knee osteoarthritis decision-making with AI	Comparative study (AI vs traditional)	AI-enabled patient decision aids significantly improve decision quality and shared decision-making in adults with knee osteoarthritis compared to traditional educational materials, enhancing both patient experience and functional outcomes
Value-Based Healthcare: Can Generative Artificial Intelligence and Large Language Models Be a Catalyst for Value-Based Healthcare? [28]	The potential of generative AI and large language models to drive value-based healthcare.	Conceptual analysis/Opinion article	Generative AI and large language models could enhance value-based healthcare by improving decision-making and optimizing patient outcomes, though challenges in implementation remain
A Collaborative Visualization Tool to Support Doctors’ Shared Decision-Making on Antibiotic Prescription [27]	AI visualization tool to support responsible antibiotic prescribing	Tool development/proof-of-concept	The collaborative visualization tool improved decision-making by enhancing communication between doctors and patients, leading to more informed antibiotic prescriptions
Patient and Family Engagement During Treatment Decisions in an ICU: A Discourse Analysis of the Electronic Health Record [29]	Enhancing documentation in SDM within intensive care settings	Applied study	Patient and family engagement in ICU treatment decisions was facilitated by electronic health record documentation, but challenges in communication and decision-making processes remain
Impact of Direct Use of Artificial Intelligence Algorithms on Patient Autonomy in Dermatology [30]	Applications of AI in dermatology	Overview or case-based discussion	The direct use of AI algorithms in dermatology can enhance diagnostic accuracy but raises concerns about patient autonomy and decision-making
Artificial Intelligence in Oncology: Chances and Pitfalls [31]	Cautious approach to AI use in oncology	Perspective/commentary	AI has the potential to revolutionize oncology by improving diagnostic precision, but challenges such as data quality and ethical concerns must be addressed
Artificial Intelligence and Decision-Making in Healthcare: A Thematic Analysis of a Systematic Review of Reviews [32]	Ethical considerations in healthcare AI	Narrative review	AI can significantly enhance decision-making in healthcare, though integration challenges and concerns about transparency and accountability persist
Artificial Intelligence in Surgical Risk Prediction [33]	AI’s potential in surgical risk prediction	Applied study/Model application	AI can improve surgical risk prediction, but further validation and integration into clinical practice are needed to ensure reliability and accuracy
Enabling Pregnant Women and Their Physicians to Make Informed Medication Decisions Using Artificial Intelligence [34]	AI-assisted decision-making for pregnancy-related drug use	Case-based or clinical scenario analysis	AI can help pregnant women and physicians make informed medication decisions, but careful consideration of safety and personalized approaches is essential
Artificial Intelligence Promotes Shared Decision-Making through Recommending Tests to Febrile Pediatric Outpatients [35]	Role and integration challenges of AI in pediatric SDM	Qualitative or mixed-methods	AI enhances shared decision-making in febrile pediatric outpatient care by recommending appropriate tests, supporting more informed treatment decisions
Artificial Intelligence and the Doctor-Patient Relationship Expanding the Paradigm of Shared Decision Making [36]	Preserving the doctor-patient relationship in AI-supported care	Perspective or conceptual commentary	AI can expand the paradigm of shared decision-making by enhancing communication and patient involvement, but its integration into the doctor-patient relationship must be carefully managed to maintain trust
The Use of Artificial Intelligence in Clinical Care: A Values-Based Guide for Shared Decision Making [37]	Values-based integration of AI in decision-making	Ethical/conceptual analysis	AI in clinical care should be guided by values-based frameworks to ensure that shared decision-making prioritizes patient values and promotes ethical outcomes
AI and the Need for Justification (to the Patient) [38]	Ensuring transparency in AI-assisted decisions	Conceptual/Position paper	AI in healthcare requires clear justification to patients to ensure transparency, trust, and alignment with ethical principles in decision-making
A Scoping Review of Knowledge Authoring Tools Used for Developing Computerized Clinical Decision Support Systems [39]	Development of accessible SDM tools using AI	Design-focused/Applied development	Knowledge authoring tools play a crucial role in developing effective clinical decision support systems, but further research is needed to improve their usability and integration in clinical practice
PROPOSE. Development and Validation of a Prediction Model for Shared Decision Making for Patients with Lumbar Spinal Stenosis [40]	“PROPOSE” model to improve SDM in spinal stenosis care	Framework/model proposal	The PROPOSE model effectively predicts the likelihood of shared decision-making in patients with lumbar spinal stenosis, aiding clinicians in personalized treatment planning
Impact of Artificial Intelligence on Prognosis, Shared Decision-Making, and Precision Medicine for Patients with Inflammatory Bowel Disease: A Perspective and Expert Opinion [41]	Benefits and challenges of AI in managing inflammatory bowel disease	Review or applied discussion	AI has the potential to enhance prognosis, shared decision-making, and precision medicine in inflammatory bowel disease, but careful integration is necessary to maximize its benefits for patients
Application of Artificial Intelligence in Shared Decision Making: Scoping Review [42]	AI’s potential to enhance patient engagement	Conceptual/Commentary	AI has significant potential to support shared decision-making in healthcare, though challenges related to data quality, trust, and integration into clinical workflows need to be addressed
Basic Principles for the Development of an AI-Based Tool for Assistive Technology Decision Making [43]	Transparency in developing assistive AI systems	Technical/conceptual study	Developing AI-based tools for assistive technology decision-making requires careful consideration of user needs, ethics, and the ability to provide personalized recommendations
Improving shared decision-making about cancer treatment through design-based data-driven decision-support tools and redesigning care paths: an overview of the 4D PICTURE project [44]	Data-driven pathways to optimize cancer-related SDM (4D PICTURE project)	Applied research project	The 4D PICTURE project demonstrates that design-based, data-driven decision-support tools can improve shared decision-making in cancer treatment by redesigning care paths to better align with patient values
The Impact of Artificial Intelligence on the Person-Centred, Doctor-Patient Relationship: Some Problems and Solutions [45]	Balancing AI utility with a patient-centered approach	Ethical/strategic commentary	AI could potentially disrupt the person-centerd doctor-patient relationship, but strategies like enhancing transparency and maintaining human oversight can help preserve trust and communication
Speech Recognition Can Help Evaluate Shared Decision Making and Predict Medication Adherence in Primary Care Setting [46]	Use of voice recognition to enhance SDM in primary care	Applied pilot or feasibility study	Speech recognition technology can be used to evaluate shared decision-making and predict medication adherence in primary care, improving patient outcomes and care quality
Artificial Intelligence in medicine: reshaping the face of medical practice [47]	Balancing AI benefits with ethical standards	Conceptual/ethical discussion	AI is reshaping medical practice by enhancing diagnostic accuracy and decision-making, but ethical challenges, including transparency and accountability, must be addressed for its effective integration
ASO Author Reflections: Enhancing Surgical Decision-Making for Breast Reconstruction – Machine Learning-Driven Prediction of Postoperative Quality of Life [48]	Machine learning applications in breast reconstruction surgery	Model application/clinical study	Machine learning models can enhance surgical decision-making for breast reconstruction by predicting postoperative quality of life, enabling more personalized patient care
Computer-Based Decision Tools for Shared Therapeutic Decision-Making in Oncology: Systematic Review [49]	Supportive role of AI in oncology SDM and gaps in training	Empirical + educational focus	Computer-based decision tools in oncology can improve shared therapeutic decision-making, but their effectiveness depends on user engagement and the integration of personalized data

## 4 Discussion

The integration of AI into SDM represents a significant advancement in healthcare, expanding the potential to enhance patient care through more precise and personalized recommendations informed by comprehensive data analysis. For instance, the multi-purpose AI health bot developed by Afsar is designed to support both doctors and patients in selecting optimal treatments, aiming to reduce doctors’ workload while empowering patients to take an active role in managing their health [11]. Similarly, the web-based decision-support tool introduced by Chen *et al.* provides women with clear, evidence-based information for choosing between vaginal birth and repeat cesarean, exemplifying AI’s capacity to assist in informed decision-making [14]. One of the core strengths of incorporating AI into SDM lies in its ability to swiftly analyze large datasets, thereby offering insights and tailored recommendations that may not be immediately evident through traditional methods. This potential is reflected in studies such as those by Jang *et al.*, which focus on assessing hip arthroplasty risks, and by Xu, which examines decision-making in breast reconstruction surgery [25,48]. However, the integration of AI in SDM presents various challenges. Research by Begley *et al.* raises the issue of “inherited defeaters” – pre-existing biases that can influence AI-driven decision-making outcomes, posing a risk of perpetuating these biases in clinical settings [12]. Addressing this challenge requires careful monitoring and frequent updates to AI systems to prevent algorithmic bias. Moreover, Holm’s study points out that while technical accuracy is essential, AI systems must also be evaluated for their effectiveness in promoting SDM that aligns with patients’ expectations and values [23]. Maintaining patient autonomy and trust in AI-assisted decision-making remains a crucial consideration. Studies by Karaa highlight concerns that AI’s role might overshadow the patient’s central position in SDM, potentially diminishing the human aspect of healthcare [30]. This sentiment is echoed by Sauerbrei *et al.*, who emphasize the need for AI to complement rather than replace human interaction, thereby preserving the core principles of patient-centered care [45]. Practical implementation challenges, including data quality, interoperability, and clinician training, are also essential factors for successful AI adoption. Nair *et al.* stress the importance of investing in accessible and interoperable tools to enhance clinical decision-support systems’ effectiveness, underscoring the need for AI solutions that seamlessly integrate into existing healthcare workflows [39]. Looking forward, the future of AI in SDM appears promising. Emerging technologies, such as generative AI and large language models, offer opportunities to further personalize care and improve decision-making efficiency, as discussed by Jayakumar *et al.* [28]. Future research and development should focus on addressing current limitations in AI systems, such as enhancing algorithmic transparency and addressing ethical considerations, as highlighted by Khosravi *et al.* and Tretter *et al.* [32,47].

## 5 Conclusion

The integration of AI into shared decision-making (SDM) marks a transformative development in healthcare, enhancing personalization and accuracy in treatment decisions. Although AI offers substantial potential to improve patient care, its effective and ethical application requires addressing several critical challenges [50–52]. Among the foremost concerns are managing inherent biases, safeguarding patient autonomy, and navigating practical implementation hurdles. As AI technology advances, efforts should focus on creating tools that reinforce the human dimension in healthcare, fostering a collaborative, person-centered approach to decision-making. Future research and development must prioritize these challenges while exploring innovative applications to unlock AI’s full potential in SDM.
